# Calendar

**DOI:** 10.1155/S1463924679000675

**Published:** 1979


### Amino acid analyser

LKB has announced the availability of their 4400 Amino acid analyser. The instrument provides fast analysis times for both protein hydrolysates (65 minutes) and physiological fluid. This performance is achieved using a horizontal, stainless steel analytical column mounted in a solid-state heating system and a high temperature reaction coil for fast colour development. It has a 70 sample, cooled autoloader and optical data-handling accessory.
*LKB Instruments Ltd, 232 Addington Road, Selsdon, South Croydon, Surrey, CR2 8YD, UK.*

### Automatic clinical analyser

An automatic clinical analyser has been developed by Du Pont. The analyser, the 'aca' III, currently under evaluation in Europe, incorporates the same characteristics as the present 'aca' instruments. It includes these additional features: computer assisted method calibration, automatic instrument standardisation, instrument diagnostic programs, expanded report format and alphanumeric keyboard and display. It has the capacity to allow new methods to be added as they are developed. Vital parameters are displayed in a selected language to aid instrument operation. The instrument is compatible with any on-line laboratory information system.
*Du Pont de Nemours Belgium SA, Instruments Products Division, Mecure Center, 1130 Brussels, Belgium.*

### Spectrophotometer

Du Pont's UV spectrophotometer, introduced at the Pittsburgh Conference on Analytical Chemistry, is a compact variable wavelength detector. The modular detector allows selection of the optimum operating wavelength specific to the absorption of the compound to be measured. When operated at the UV absorption maximum of the compound being measured, sample detection sensitivity can be increased.

The spectrophotometer incorporates a stable air-cooled deuterium source and a high efficiency dual beam grating monochrometer. Absorbance ranges are adjustable from 0.005 to 2.56 AUFS in ten steps. The unit complements their 850 and 860 HPLC systems and it is compatible with most liquid chromatographs.
*Du Pont (UK) Ltd, 64a Wilbury Way, Hitchin, Herts SG4 OUR, UK.*

### Automatic melting points

An automatic melting point instrument, the Mettler FP61, is available from MSE Scientific Instruments. It is portable and simple to use. The melting point is indicated on a digital display readable to 0.1 °C and the melting process can be graphically reproduced by a recorder connected to the instrument.
*MSE Scientific Instruments, Manor Royal, Crawley, Sussex, UK.*

### Calendar

**Editor's Note:**
***Organisers of conferences, seminars etc. should send details for inclusion in this calandar as soon as the relevant information is available and not later than three months before the event.***

**Synthesis in Organic Chemistry.**
July 23-26, Cambridge, UK.
*Dr. M. Robinson, The Chemical Society, Burlington House, London W1, UK.*

**3rd International Conference on the Chemistry and Uses of Molybdenum.**
August 19 - 23, Michigan
*Climax Molybdenum Co. Ltd., 1 - 3 Grosvenor Place, London SW1, UK.*

**1st European Symposium on Organic Chemistry.**
August 20 - 23, Cologne, W. Germany
*Dr. W. Fritsche, c/o Gesellschaft Deutscher Chemiker, PO Box 90 04 40, D-6000 Frankfurt, Main 90, FRG.*

**5th Australian Symposium on Analytical Chemistry.**
August 20 - 24, Perth, Australia
*B. Codling, c/o Government Chemical Laboratories, 30 Plain Street, Perth, WA 6000, Australia.*

**Automatic methods in connection with Trace-organic Analysis**
September 4 - 7, Guildford, U.K.
*Dr. E. Reid, Wolfson Bioanalytical Centre, University of Surrey, Guildford GU2 5XH, U.K.*

**Automation and Computerisation in the Medical Laboratory**
September 5 - 7, Stirling University, Scotland.
*G.W. Thomson, Secretary, IMLS Glasgow Branch, 67 Glen Oglivie, St. Leonards, East Kilbride, Scotland.*

**Management Studies for R & D Chemists.**
September 10 - 14, Slough, UK.
*Mrs. S. Leclercq, The Chemical Society, Burlington House, London W1V OBN, UK.*

**Laboratory 79**
September 11-13, London, UK.
*Curtis Steadman, 26-34 High Street, Saffron Waldon, Essex CB10 1EP, UK.*

**International Conference on Flow Analysis**
September 11 - 13, Amsterdam, The Netherlands.
*Secretary FA-Amsterdam,. Laboratory for Analytical Chemistry, University of Amsterdam, Nieuwe Achtergracht 166, 1018 WV Amsterdam, The Netherlands.*

**Communications in Microprocessor Industrial Instrumentation**
September 12 - 13, London U.K.
*Mrs. P. Keiller, Sira Institute, South Hill, Chiselhurst, Kent BR7 5EH, U.K.*

**Ninth International Meeting on Organic Geochemistry**
September 17 - 20, Newcastle-upon-Tyne, U.K.
*Dr.A.G. Douglas, Organic Geochemistry Unit, Geology Department, Drummond Building, The University, Newcastle-upon-Tyne, NE1 7RU, U.K.*

**Colloquium: Apport de l'informatique a l'analyse industrielle pour le controle et la conduite des procedes.**
September 18 - 19, Lyon
*Societe de Chimie Industrielle, 28, rue Saint-Dominique, 75007 Paris, France.*

**Automatic on-line industrial chromatograph: sophisticated analyser or industrial transducer? – colloquium.**
October 3 - 5, Arles
*Le Comite d'Organisation, Institut de regulation et automation, Chemin des Moines, 13644 Arles, France.*

**Real-time Datahandling and Process Control**
October 23 - 25, Berlin, West Germany
*Congress Organisation Company, John Foster Dulles Allee 10, D-1000 Berlin, West Germany.*

**International Liquid Chromatography Symposium IV.**
October 24 - 25, Strasbourg, France.
*X Delemere, Waters Associates SA, 18 rue Goubet, 75019 Paris, France.*

**1980**

**Automation at SAC 80**
July 20 - 26, Lancaster, U.K.
*The Secretary, Analytical Division, The Chemical Society, Burlington House, London, W1Y OBN, U.K.*

**1981**

**Euroanalysis IV**
August 23 - 28, Helsinki, Finland.
*Association of Finnish Chemical Societies. Executive Secretary, Pohj, Hesperiankatu 3B10, SF-00260 Helsinki 26, Finland.*

